# Advances in research on the relationship between mitochondrial dysfunction and osteoporosis: a bibliometric study from 2014 to 2024

**DOI:** 10.3389/fmed.2025.1597116

**Published:** 2025-07-03

**Authors:** Yun-song Zhang, Jia-nan Yu, Ming-ze Han, Bin Si, Zhen-hua Li, Ji-cheng Han

**Affiliations:** ^1^School of Integrated Traditional Chinese and Western Medicine, Changchun University of Chinese Medicine, Changchun, China; ^2^School of Traditional Chinese Medicine, Changchun University of Chinese Medicine, Changchun, China; ^3^Affiliated Hospital of Changchun University of Chinese Medicine, Changchun, China

**Keywords:** mitochondria, osteoporosis, oxidative stress, bone homeostasis, mitochondrial metabolism

## Abstract

**Introduction:**

Osteoporosis, characterized by reduced bone mineral density and increased fracture risk, poses a major health challenge in aging populations. Emerging evidence indicates that mitochondrial dysfunction plays a crucial role in its pathogenesis, though a comprehensive analysis of research trends and therapeutic potential is lacking.

**Methods:**

We conducted a bibliometric analysis of 780 articles from the Web of Science Core Collection (2014–2024) using CiteSpace and VOSviewer to visualize research trends, collaboration networks, and emerging hotspots.

**Results:**

Annual publications showed a significant upward trend, with China and the United States as leading contributors. Key journals (e.g., Journal of Biological Chemistry, Nature) and core themes were identified: oxidative stress (177 occurrences), apoptosis, mitophagy, and mitochondrial transfer. Cluster analysis revealed emerging frontiers, including ferroptosis and SIRT1 signaling pathways, with rapid citation growth. Interdisciplinary linkages highlighted connections between mitochondrial quality control, redox balance, and bone metabolism.

**Discussion:**

Therapeutic strategies targeting oxidative stress (e.g., SIRT1 activators, vitamin K2, nanoparticle-based interventions) showed preclinical promise in restoring bone homeostasis. Mitochondrial transfer mechanisms and ferroptosis inhibitors were proposed as novel approaches for bone defect repair and diabetic osteoporosis management. This study provides new molecular insights and future directions for osteoporosis prevention and treatment.

## 1 Introduction

Osteoporosis (OP) is a systemic skeletal disorder characterized by decreased bone density, loss of skeletal integrity and increased susceptibility to fracture (1). According to statistics, the risk of vertebral fractures caused by OP increases by five times, and the risk of fractures in other parts increases by two to three times (2). This disease poses significant threats to human health, including pain, disability, and elevated mortality and also bring about enormous social and economic burdens (3, 4). At present, the number of OP patients worldwide has reached as high as 200 million. Considering the trend of population aging, it is estimated that by 2050, the proportion of the world’s population aged 60 or above will climb to 22% (5). However, the pathogenesis of OP has not been fully elucidated, and effective preventive and curative measures are still lacking.

Mitochondria are double-membrane-bound organelles found in the cytoplasm of most eukaryotic cells. They serve as the powerhouses of the cell, generating adenosine triphosphate (ATP), which is the cell’s primary energy currency (6). Under normal physiological conditions, cells maintain a complex regulatory network through mitochondrial fusion and fission, autophagy, and apoptosis, which influences mitochondrial quality control. This network is crucial for maintaining mitochondrial homeostasis and ensuring proper cellular function. Recent studies have shown that the imbalance of mitochondrial quality control is widely involved in the occurrence and development of osteoarthritis, osteoporosis and osteosarcoma (7, 8). Mitochondrial fusion fission disorders may cause abnormal distribution and dysfunction of mitochondria in skeletal muscle cells, and then affect the normal function of skeletal muscle and bone metabolism homeostasis (9). In particular, the decrease of bioenergy and the accumulation of reactive oxygen species (ROS) caused by mitochondrial dysfunction are considered to be the key causes of aging of bone marrow mesenchymal stem cells (BMSCs) (10). In recent years, the link between mitochondrial dysfunction and osteoporosis has received increasing attention. Its dynamical imbalance triggers oxidative stress, regulates cell apoptosis, and impacts osteocyte energy metabolism, and its impact on bone homeostasis and related molecular mechanisms and signaling pathways has become a hot research topic. However, the molecular mechanisms underlying these processes remain insufficiently elucidated,” and the treatment strategies for mitochondrial dysfunction are still limited.

Bibliometrics is a branch of informatics. It refers to the in-depth analysis of the bibliometric characteristics of the literature system, combined with quantitative and qualitative analysis methods, in order to achieve accurate quantitative description of the distribution, correlation and clustering status of the research field (11). The research content of bibliometrics covers many dimensions such as the contribution and influence evaluation of different authors, countries/regions, research institutions, discipline branches and journals in the academic field, and makes an in-depth analysis of the status, development trend and frontier dynamics of research activities. Additionally, analyzing data and visualizing results with VOSviewer, CiteSpace, and similar bibliometric visualization tools (12–14). It has become one of the popular techniques for evaluating the credibility, quality, and impact of academic work. Until now, there have been no bibliometric studies on mitochondrial dysfunction and OP. To fill this gap, this study is based on the Web of Science^™^ Core Collection (WoSCC). It searches for relevant bibliometric data in the T cell and AS research fields, such as annual articles, countries/regions, authors, institutions, disciplines, journals, references, and keywords, and performs descriptive statistics. This paper discusses the current status, hotspots, and frontiers of T cell and AS research. It also uses CiteSpace and VOSviewer to create knowledge maps, aiming to provide a reference for future related studies.

## 2 Materials and methods

### 2.1 Search strategy and data sources

**Figure 1:**
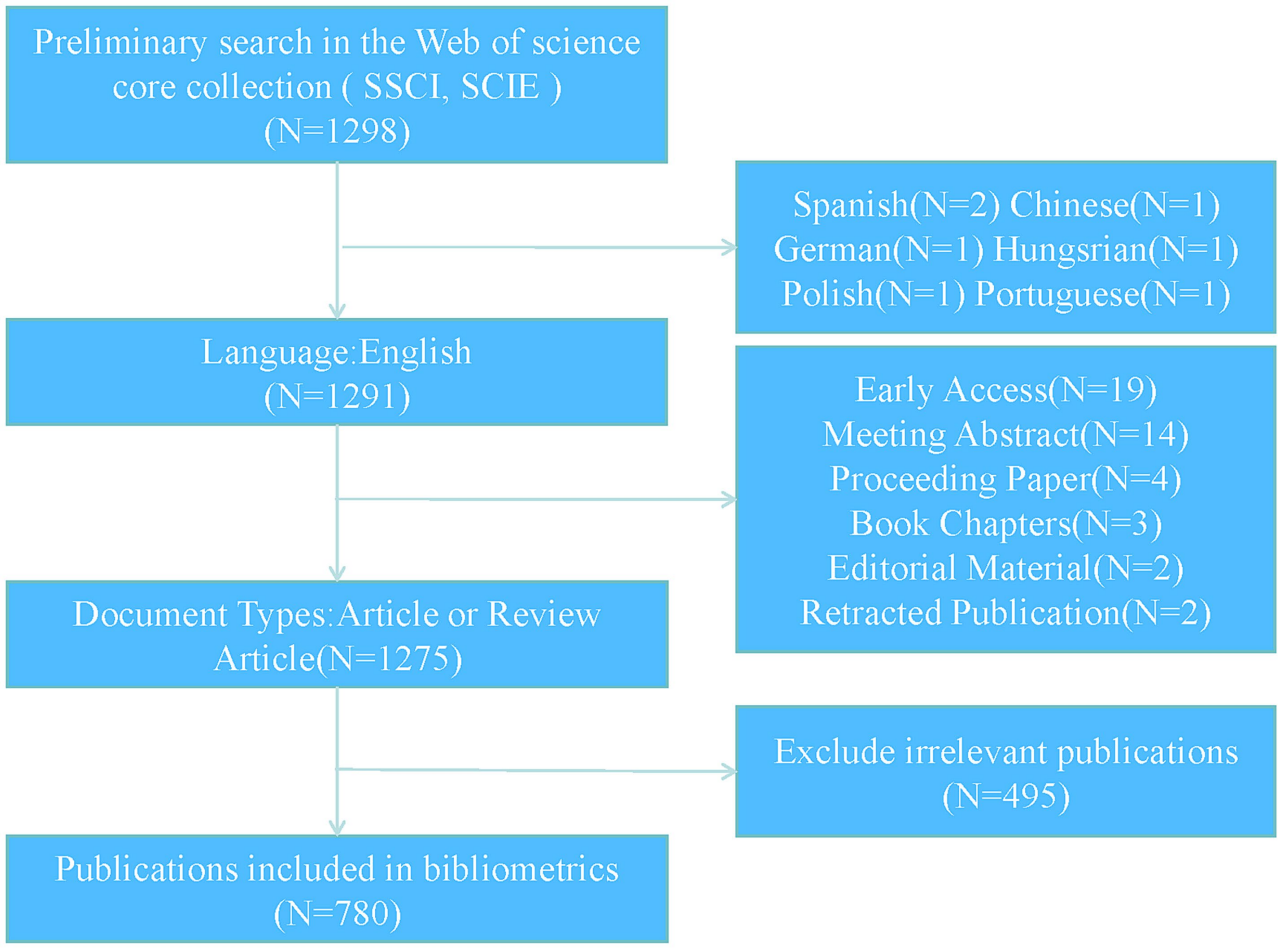
PRISMA flow chart for study selection.

To ensure the comprehensiveness and credibility of the analyzed data, the Web of Science (Core Collection) was chosen as the data source, with the indexes SCI-Expanded and SSCI selected. The search strategy employed was: “TS = (osteoporosis OR osteopenia OR osteoporotic OR bone loss OR low bone mass OR low bone density) AND TS = (Mitochondrion OR Mitochondria OR Mitochondrial).” The search was conducted within the time frame of January 1, 2014, to November 5, 2024. The search was restricted to articles and reviews published in English, while other types of publications, such as meeting abstracts, conference papers, and editorial materials, were excluded. The screening process was independently conducted by two authors to ensure the reliability of the included publications. Any discrepancies between the authors were resolved through discussion and consensus. The full records and cited references of the included publications were exported in plain text format. The specific search strategy and screening process are illustrated in Figure 1.

### 2.2 Analysis methods

To conduct bibliometric analysis, three software tools—CiteSpace, VOS viewer, and the “Bibliometrix” package—are utilized. Each of these tools has its own strengths in the field of bibliometric analysis.

CiteSpace is adept at creating visual maps of scientific knowledge, which intuitively present the knowledge structure and development trends within a particular field. The core functions of CiteSpace include generating unique co-citation networks composed of multiple document co-citation networks, as well as automatically producing related analytical results. Each network corresponds to a specific time slice, revealing the overall structure of the network, clustering within the network, associations between clusters, key nodes, and pathways, among other features.

VOS viewer excels in generating any type of textual map and offers a variety of charts and visualizations to assist researchers in analyzing, visualizing, and understanding academic literature, knowledge networks, collaborative relationships, and research hotspots. It is particularly useful for its clear and intuitive graphical representations that can facilitate the interpretation of complex data.

The “Bibliometrix” package is an R-based software package for bibliometric analysis and scientific visualization. It provides a suite of tools for the quantitative study of scientific literature, enabling researchers to perform in-depth analyses of publication patterns, citation distributions, and other bibliometric indicators. This package supports a wide range of bibliometric studies by offering comprehensive functionalities for data processing, statistical analysis, and visualization.

## 3 Results

### 3.1 Articles basic information and publication trends

**Figure 2:**
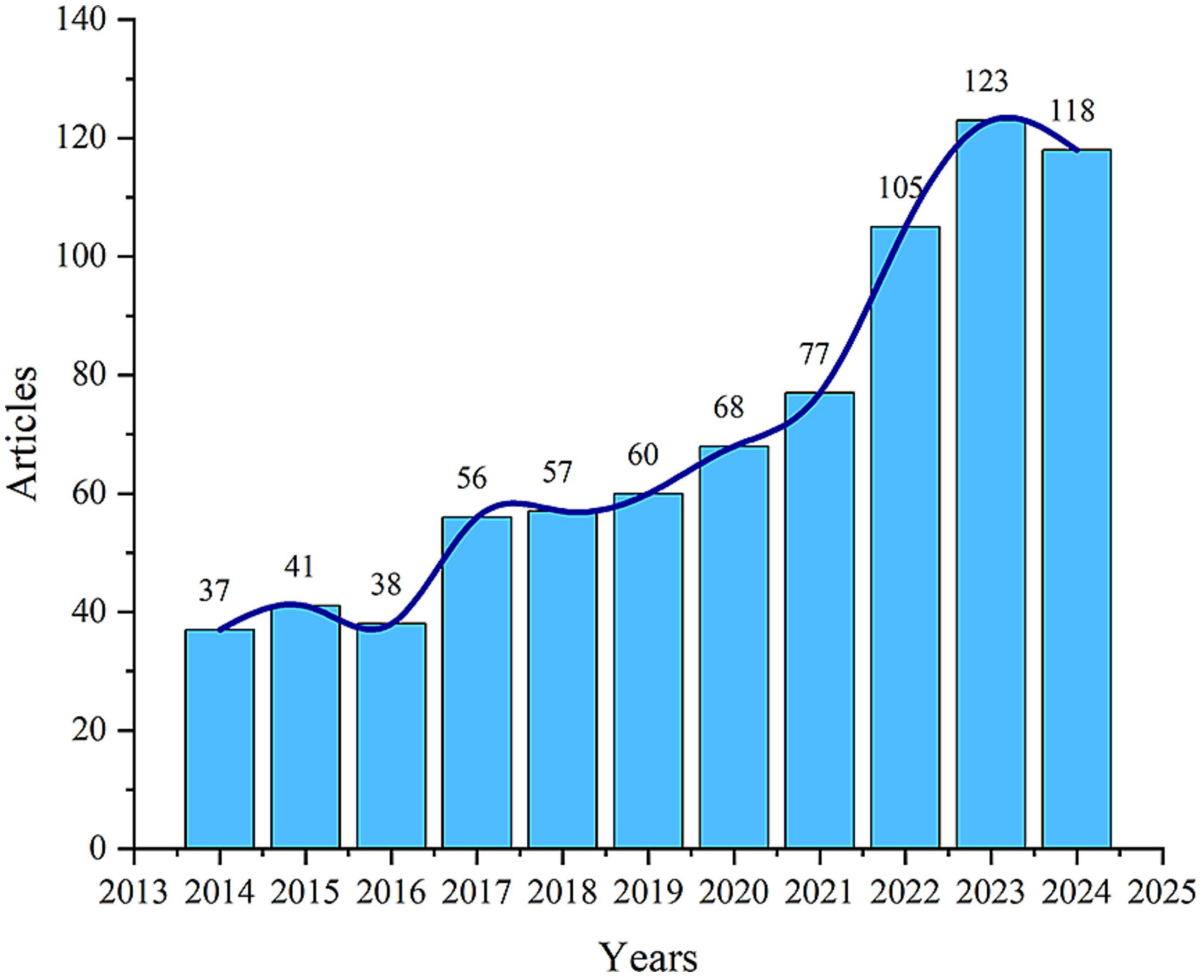
Annual and cumulative growth trend of publications.

The 780 articles used in this study were authored by 5,071 researchers from 1,171 organizations across 60 countries, published in 328 journals, and cited 42,976 references from 4,454 different journals. Figure 2 shows the temporal distribution of publications related to mitochondrial dysfunction and osteoporosis. Overall, the number of publications in this field has shown an upward trend. Notably, there was a significant increase in the number of publications after 2016, with annual publication output surpassing 100 articles since 2022. This phenomenon indicates that this field has garnered escalating scholarly interest in recent years, becoming a hot topic within the fields of osteoporosis and bone metabolism.

### 3.2 Bibliometric analysis of authors

**Figure 3:**
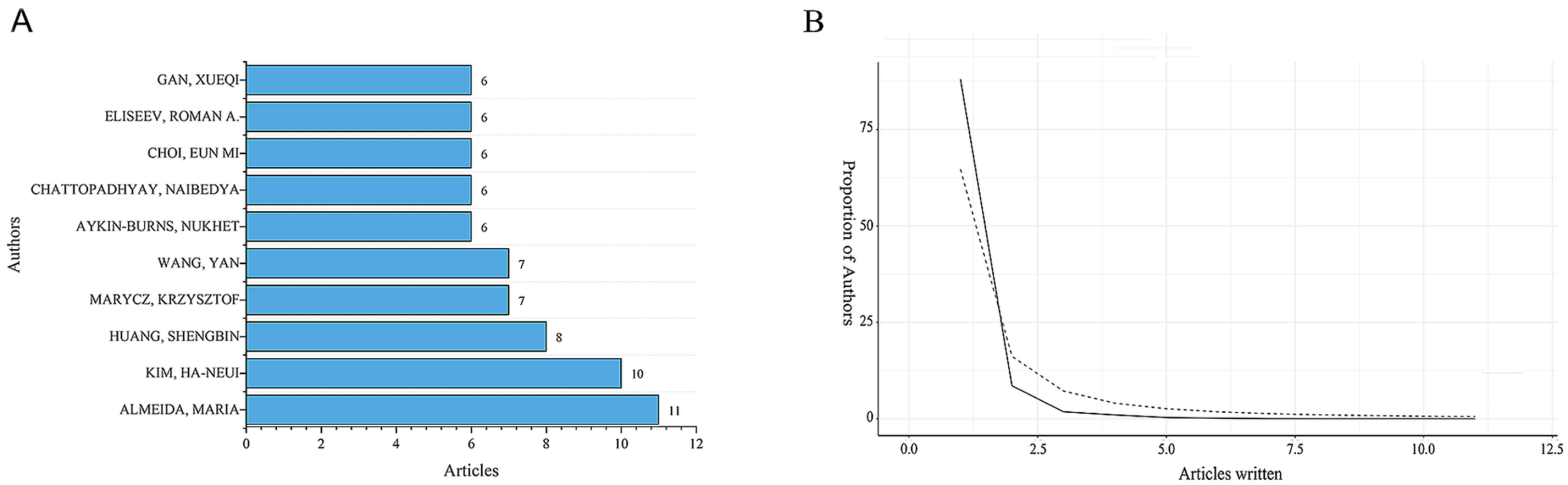
Distribution of publications and citations from different authors. **(A)** Top 10 authors with the most publications. **(B)** Lotka’s Law of publication authorship.

By conducting a bibliometric analysis of the authors of these articles, it is possible to identify representative scholars and core research forces in the field. Table 1 and Figure 3A show the top 10 most productive authors in terms of publication output within this research field. According to the well-known scholar Derek J. de Solla Price, in any given research field, approximately half of the articles are authored by a group of highly productive authors. The size of this group is roughly equal to the square root of the total number of authors in the field, i.e.,


$$\sum_{m+1}^{1}n(X)=\sqrt{N}$$

*n* (*X*) denotes the number of authors writing *X* articles, *I* = *n*_max_ denotes the number of articles of the most productive authors in the field, *N* denotes the total number of authors, and *m* denotes the minimum number of publications of core authors.

**Table 1:** Most relevant top 10 authors.

Rank	Author	Articles	Citations	Average citation/publication
1	Almeida, Maria	11	803	73
2	Kim, Ha-Neui	10	509	50.9
3	Huang, Shengbin	8	283	35.38
4	Marycz, Krzysztof	7	95	13.57
5	Wang, Yan	7	261	37.29
6	Aykin-Burns, Nukhet	6	177	29.5
7	Chattopadhyay, Naibedya	6	125	20.83
8	Choi, Eun MI	6	100	16.67
9	Eliseev, Roman A	6	119	19.83
10	Gan, Xueqi	6	156	26

The statistical information shows that in this research field, *n*_max_ = 7. According to Price’s Law, authors who have published more than 3 articles are considered core authors. In total, there are 176 core authors who have collectively published 664 articles, accounting for 85.1% of the total number of publications. This meets the criteria proposed by Price, indicating that a relatively stable group of collaborating authors has been established in this field. However, among the top 10 authors who contributed to the publications in the field of mitochondrial and OP research, the top 10 authors accounted for 12.8% of the total number of publications. This level of concentration may imply a “Matthew effect” in academic resource allocation, where a small number of high-output authors dominate the research direction. And there is insufficient participation of emerging scholars, resulting in the slow development of new research topics due to inadequate authoritative support. In this field, the proportion of authors with more than one publication also conforms to Lotka’s Law (Figure 3B). Among them, Almeida has the highest number of publications. From January 2004 to November 2024, he published 11 papers, which received a total of 803 citations, with an average of approximately 73 citations per paper. Nevertheless, the core authors’ collaboration network is primarily based on domestic cooperation, with only 21% of the collaboration being international. This “localised” cooperation pattern may hinder the innovation of cross-cultural research design.

### 3.3 Bibliometric analysis of journals

**Figure 4:**
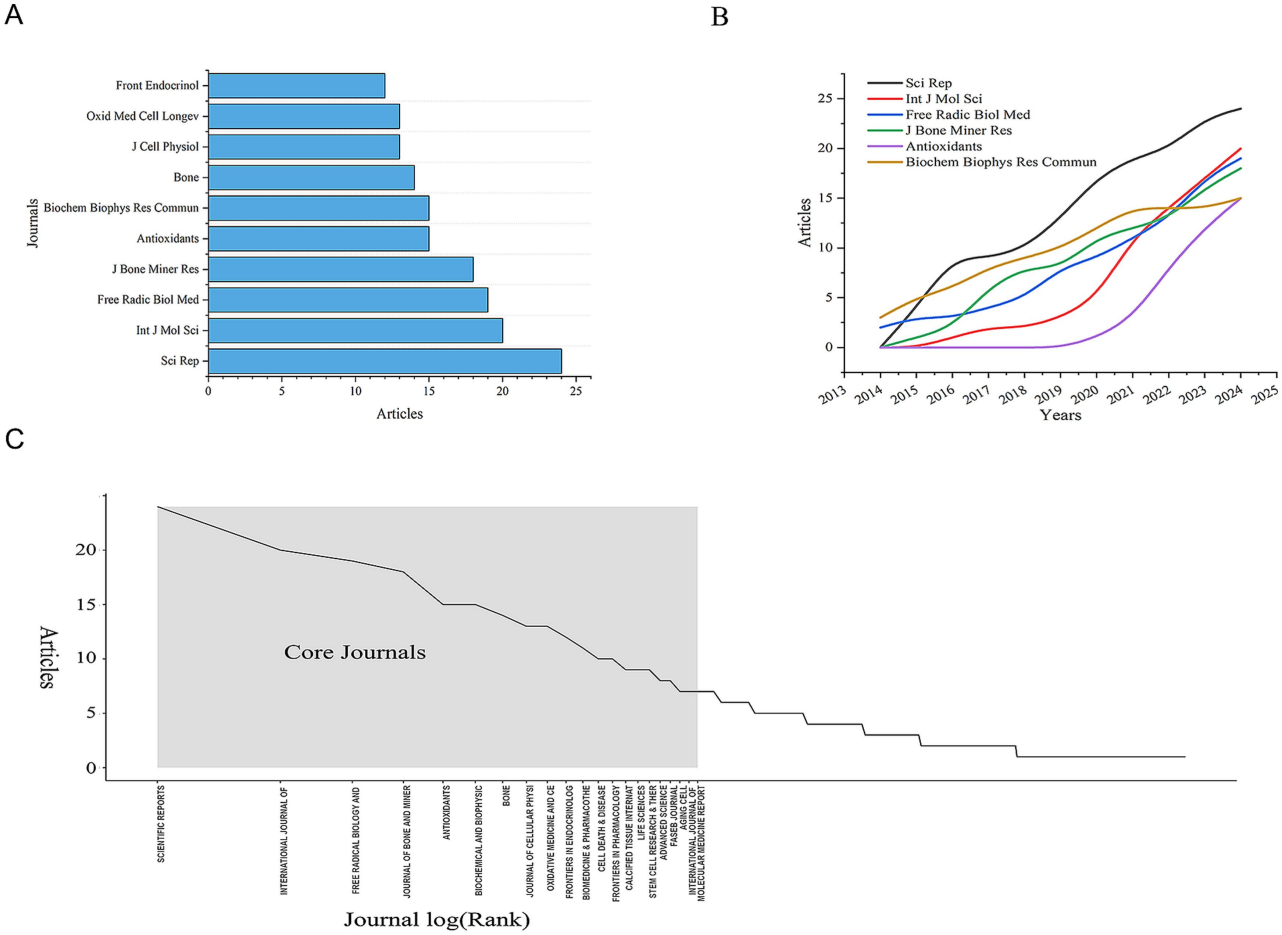
Distribution of publications and citations from different journals. **(A)** Top 10 journals with the most publications. **(B)** Annual and cumulative growth trends in journals. **(C)** Core journals.

All 780 articles were published across 328 journals. After analyzing the publication counts and citation frequencies of all journals, we found that the journal with the highest number of publications is Scientific Reports, followed by International Journal of Molecular Sciences, with 24 and 20 articles, respectively (Table 2 and Figure 4A). Both of these journals are open-access, indicating that the development of open-access journals has significantly contributed to research progress in this field. This also reflects the growing acceptance among scholars of the principle of free access to research findings. But it also raises concerns about the quality of papers. Some journals’ fast-track reviews may come at the cost of rigour.

**Table 2:** Most relevant top 10 journals.

Rank	Journal	Articles	Citations	Average citation/publication
1	Scientific Reports	24	634	26.42
2	International Journal of Molecular Sciences	20	549	27.45
3	Free Radical Biology and Medicine	19	627	33
4	Journal of Bone and Mineral Research	18	583	32.39
5	Antioxidants	15	247	16.47
6	Biochemical and Biophysical Research Communications	15	383	25.53
7	Bone	14	285	20.36
8	Journal of Cellular Physiology	13	602	46.31
9	Oxidative Medicine and Cellular Longevity	13	303	23.31
10	Frontiers in Endocrinology	12	191	15.92

The 780 articles in this study collectively cited references from 4,454 different journals. Among these, the journals that contributed more than 900 cited references are Journal of Biological Chemistry, Journal of Bone and Mineral Research, and Nature (Figure 4B). The high number of cited articles from these journals reflects their authority in the field, indicating that the research published in these journals is widely recognized and referenced by scholars. Therefore, we conclude that the research findings published in these three journals are of significant interest to researchers in this field. But since journals are mostly dominated by western countries, their review preferences may indirectly shape the research paradigm in the field.

Meanwhile, in our bibliometric analysis of journals, we applied Bradford’s Law to describe the distribution of publications across different journals. We identified 21 core journals, which are considered the primary journals preferred by researchers in this field (Figure 4B). Additionally, we analyzed the annual publication output of the top 10 core journals and found that the number of publications in these journals has shown an upward trend over the years. This trend aligns closely with the overall increase in the annual number of publications in the field, reflecting that research on the mechanisms of mitochondrial dysfunction in osteoporosis has become a significant focus for scholars (Figure 4C). Notably, among core journals, only Frontiers in Endocrinology and Oxidative Medicine and Cellular Longevity explicitly focus on clinical translation, indicating a disconnection between basic and clinical research. This suggests that researchers need to pay more attention to interdisciplinary cooperation to better link basic scientific research with clinical practice.

### 3.4 Bibliometric analysis of countries

**Figure 5:**
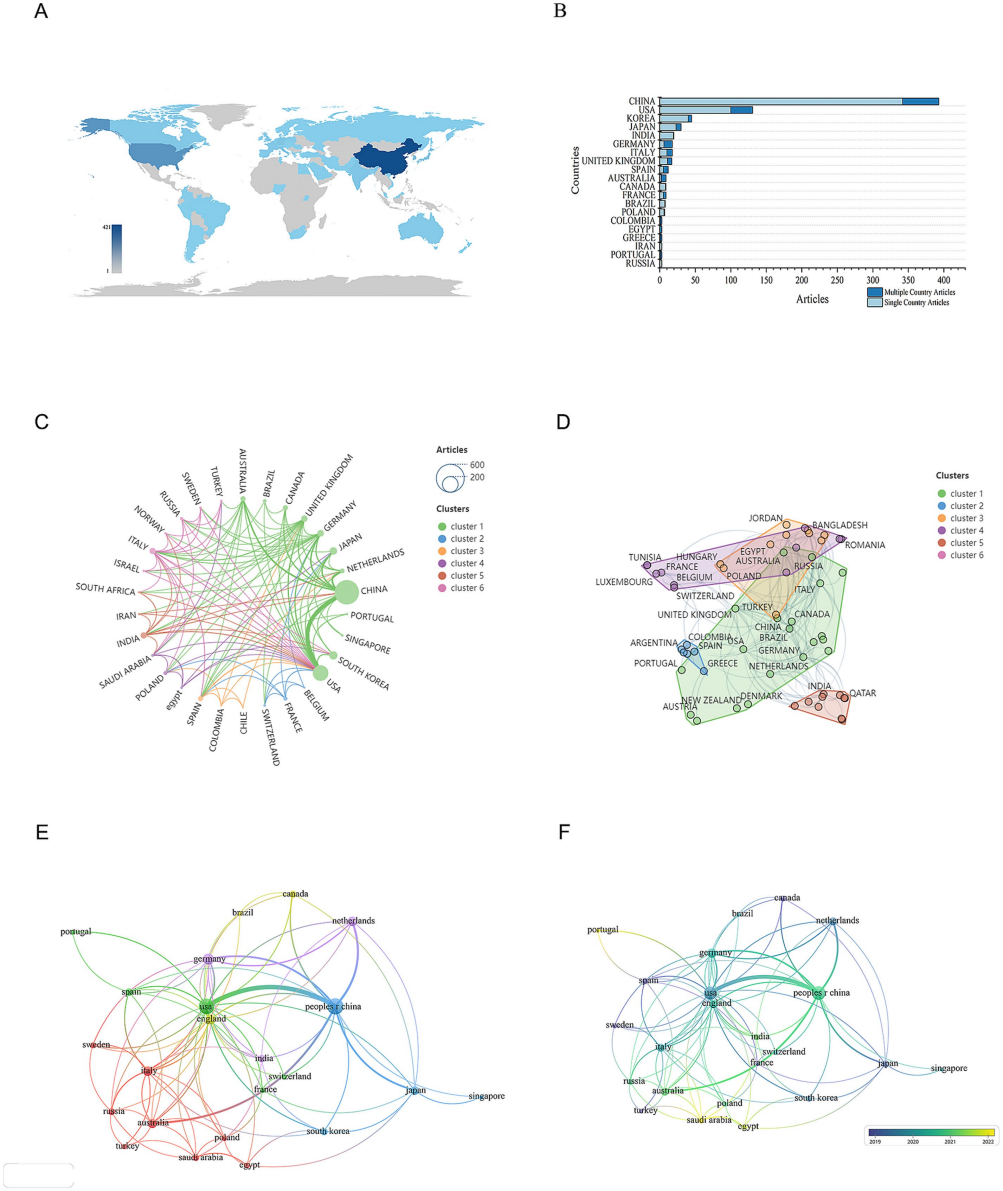
Distribution of publications and citations from different countries. **(A)** Geographic distribution map based on the total volume of publications for different countries/areas. **(B)** Top 20 countries with the most publications. **(C)** Global collaboration network in academic publications. The lines connecting the nodes represent collaborations between countries, while the colors of these lines indicate different research clusters. **(D)** Global collaboration network in research by country clusters. Each node represents a country, and the size of the node reflects its research output or contribution. The lines connecting the nodes indicate collaborative relationships between countries. **(E)** Visualization map of countries/areas citation networks generated using the VOS browser. The thickness of the lines reflects the strength of the citations. **(F)** Timeline diagrams of countries/areas citation network.

To identify which countries have made the most significant contributions to research on mitochondrial dysfunction and osteoporosis, we conducted a visual analysis of publication counts from 60 countries. In Figure 5A, darker colors indicate a higher number of publications. China leads with the highest number of publications, totaling 421 articles, followed by the United States with 173 articles. This geographical distribution highlights the uneven global allocation of scientific resources. China’s high output likely stems from its strategic investments in biomedicine, an aging population, and a growing OP disease burden. The United States, a traditional research powerhouse, excels in interdisciplinary collaboration and mature translational medicine platforms, with high-impact journals like the Journal of Bone and Mineral Research making significant contributions. Next, we performed a visual analysis of the collaboration network between countries. The results show a highly uneven distribution of publications, with a pronounced top-heavy effect, where the majority of publications are authored by scholars from a few countries (Figure 5B). This “top-heavy effect” might also result in a narrow research perspective. Current research mainly focuses on European, American, and East Asian populations, while neglecting the unique genetic diversity and environmental factors of regions like Africa and South America, which are crucial for understanding OP pathology. This limits the generalizability of the research outcomes. In terms of international collaboration, there is relatively close cooperation between countries (Figure 5C). Overall, a cooperative network centered around China and the United States has been established (Figures 5D,E). However, the relatively low participation of developing countries has intensified regional imbalances, hindering resource-limited regions from effectively addressing the public health challenge of OP. Nevertheless, India and Portugal have emerged as rising nations in this field in recent years. They are expected to achieve greater growth in the future, make up for the current research deficiencies, and become new research hubs (Figure 5F) (see Table 3).

**Table 3:** Most relevant top 10 countries.

Rank	Counties	Articles	Citations	Average citation/publication
1	China	421	9,615	22.84
2	USA	173	7,381	42.66
3	Korea	48	1,162	24.21
4	Japan	39	776	19.90
5	India	31	986	31.81
6	Germany	29	853	29.41
7	Italy	28	348	12.43
8	United Kingdom	27	896	33.19
9	Spain	19	767	40.37
10	Australia	19	497	26.16

### 3.5 Bibliometric analysis of organizations

**Figure 6:**
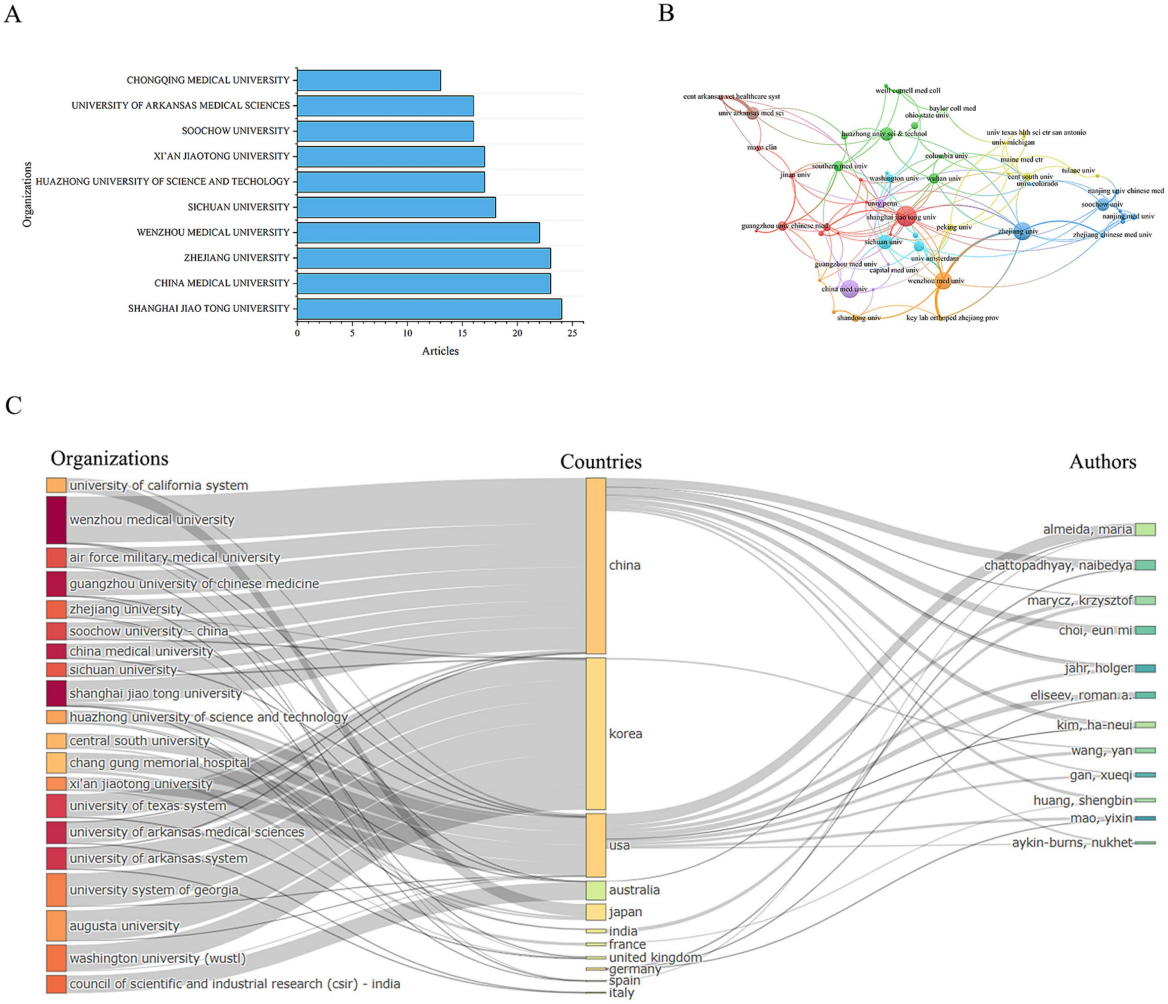
Distribution of publications and citations from different organizations. **(A)** Top 10 organizations with the most publications. **(B)** Visualization map of organizations citation networks. **(C)** Organization-country-author collaboration network map.

We conducted a statistical analysis to identify the most prominent research organizations contributing to the field of mitochondrial dysfunction and osteoporosis. The authors of all articles come from 1,171 research organizations. Among these, Shanghai Jiao Tong University has the highest publication count with 46 articles, followed by China Medical University and Zhejiang University, each with 23 articles (Table 4 and Figure 6A). The top three institutions in terms of publication output are all from China, highlighting China’s central role in this research field. However, this concentration can also lead to homogenization of research topics and insufficient exploration of emerging directions.

**Table 4:** Most relevant top 10 organizations.

Rank	Organizations	Articles	Citations	Average citation/publication
1	Shanghai Jiao Tong University	24	654	27.25
2	China Medical University	23	788	34.26
3	Zhejiang University	23	250	10.87
4	Wenzhou Medical University	22	455	20.68
5	Sichuan University	18	532	29.56
6	Huazhong University of Science and Technology	17	623	36.65
7	Xi’an Jiaotong University	17	466	27.41
8	Soochow University	16	491	30.69
9	University of Arkansas Medical Sciences	16	752	47.00
10	Chongqing Medical University	13	234	18.00

Subsequently, we performed a visual analysis of the collaboration network between research institutions. The results show that there is very close collaboration between organizations, forming a mature and well-established network, which is particularly evident in China (Figure 6B).

Furthermore, we conducted an association analysis of the leading countries, institutions, and authors in this field. The results indicate that most research outcomes are associated with institutions and authors from China and the United States. The relationships between countries, institutions, and authors are complex (Figure 6C), suggesting that international cooperation and academic exchanges in this field are highly developed. This robust international collaboration has significantly boosted the vitality and progress of the field, driving its advancement and development. However, the participation of institutions in other regions is extremely low. This “core-periphery” structure may intensify geographical bias in knowledge production. Also, the insufficient collaboration on clinical translation highlights a disconnect between industry and academia.

### 3.6 Bibliometric analysis of co-cited

The 780 articles in this study collectively cited references from 4,454 different journals. The top three journals with the highest number of cited articles are Journal of Biological Chemistry, Journal of Bone and Mineral Research, and Nature (Table 5). The volume of cited articles reflects the authority of these journals in the field, indicating that the research published in these journals is widely recognized and referenced by scholars. Therefore, we conclude that the research findings published in these three journals are of significant interest to researchers in this field.

**Table 5:** Most relevant top 10 cited journals.

Rank	Author	Citations	JCR quartile	Impact factor
1	Journal of Biological Chemistry	1,186	Q2	4.00
2	Journal of Bone and Mineral Research	1,101	Q1	6.50
3	Nature	946	Q1	69.50
4	PLoS One	857	Q2	3.75
5	Bone	842	Q1	6.3
6	Cell	809	Q1	66.85
7	Proceedings of the National Academy of Sciences of the United States of America	772	Q1	12.78
8	International Journal of Molecular Sciences	659	Q2	6.21
9	Free Radical Biology & Medicine	638	Q1	8.50
10	Scientific Reports	616	Q1	5.92

**Figure 7:**
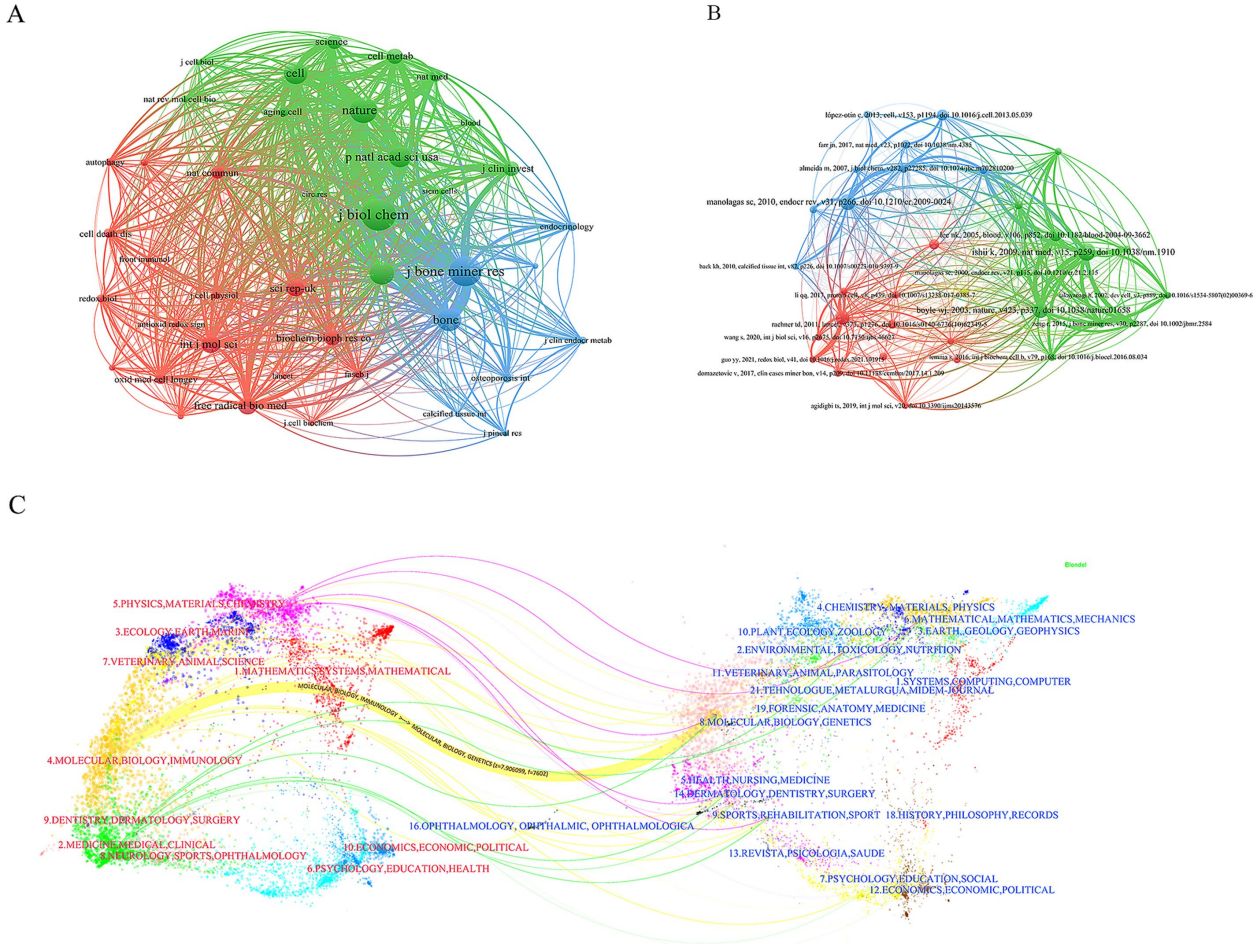
Co-citation network of publications. **(A)** Journals co-citation network. **(B)** Publications co-citation network. **(C)** Dual-map overlay results show the distribution, citation trajectory of papers between disciplines. On the left is the sizing map, on the right is the cited map, and the curves are citation links, the width of which corresponds to the intensity of the cross-citation.

Subsequently, we conducted a cluster analysis of the cited journals. The cluster analysis diagram, through its layout of nodes and edges, clearly illustrates the citation relationships and disciplinary distributions among different scientific journals. This helps to understand the interactions and influence of various journals within scientific research. The cited journals can be distinctly divided into three clusters (green, blue, and red). The green cluster includes journals such as Science, Nature, and Cell, which have strong influence in the biological sciences. The blue cluster includes journals such as Journal of Bone and Mineral Research and Bone, which have significant influence in the fields of medicine and orthopedics. And the red cluster includes journals such as Autophagy, Cell Death & Disease, Frontiers in Immunology, and Redox Biology, representing journals in the fields of biology, immunology, cell biology, and oxidative stress (Figure 7A).

The 780 articles in this study collectively cited 42,976 references. After analyzing the top 10 most frequently cited references, we found that the majority of these references are research articles, with content primarily focused on the mechanisms of mitochondrial dysfunction in the pathogenesis of osteoporosis (Table 6). We also observed that review articles from high-impact journals tend to attract significant attention, leading to higher citation counts. Subsequently, we conducted a cluster analysis of the references. The references can be clearly divided into three main clusters (red, green, and blue). Among them, the articles on red clustering focus on the fields of stem cell biology and cell death, while the articles on green clustering mainly include the fields of natural medicine and basic research, while the articles on blue clustering mostly play an important role in the fields of cell biology and molecular biology (Figure 7B).

**Table 6:** Most relevant top 10 cited references.

Rank	Author	Citations
1	Ishii K, 2009, Nat Med, V15, P259, DOI: 10.1038/NM.1910	54
2	Manolagas SC, 2010, Endocr Rev, V31, P266, DOI: 10.1210/ER.2009-0024	50
3	Boyle WJ, 2003, Nature, V423, P337, DOI: 10.1038/NATURE01658	49
4	Gao J, 2018, Cell Death Differ, V25, P229, DOI: 10.1038/CDD.2017.144	43
5	Lee NK, 2005, Blood, V106, P852, DOI: 10.1182/BLOOD-2004-09-3662	40
6	López-Otín C, 2013, Cell, V153, P1194, DOI: 10.1016/J.CELL.2013.05.039	34
7	Almeida M, 2007, J Biol Chem, V282, P27285, DOI: 10.1074/JBC.M702810200	31
8	Chen CT, 2008, Stem Cells, V26, P960, DOI: 10.1634/STEMCELLS.2007-0509	31
9	Rachner TD, 2011, Lancet, V377, P1276, DOI: 10.1016/S0140-6736(10)62349-5	27
10	Farr JN, 2017, Nat Med, V23, P1072, DOI: 10.1038/NM.4385	26

The dual-map overlay in CiteSpace is a powerful tool that not only helps us better understand the structure and evolution of citation patterns in academic literature but also provides researchers with deep insights into the dynamic changes within a specific field. In the dual-map overlay, the red labels on the left represent the research areas of the journals where the published papers are from, while the blue labels on the right represent the research areas of the journals where the cited papers are from. It is evident that most of the published papers fall within the fields of Molecular Biology and Immunology, whereas papers in the field of Molecular Biology and Genetics have the highest citation frequency (Figure 7C).

We believe that promoting interdisciplinary research in future studies will further advance the development of research on osteoporosis and mitochondrial dysfunction. By fostering cross-disciplinary collaboration, we can integrate knowledge and methodologies from different fields, leading to more comprehensive and innovative findings.

### 3.7 Bibliometric analysis of keywords and terms

**Figure 8:**
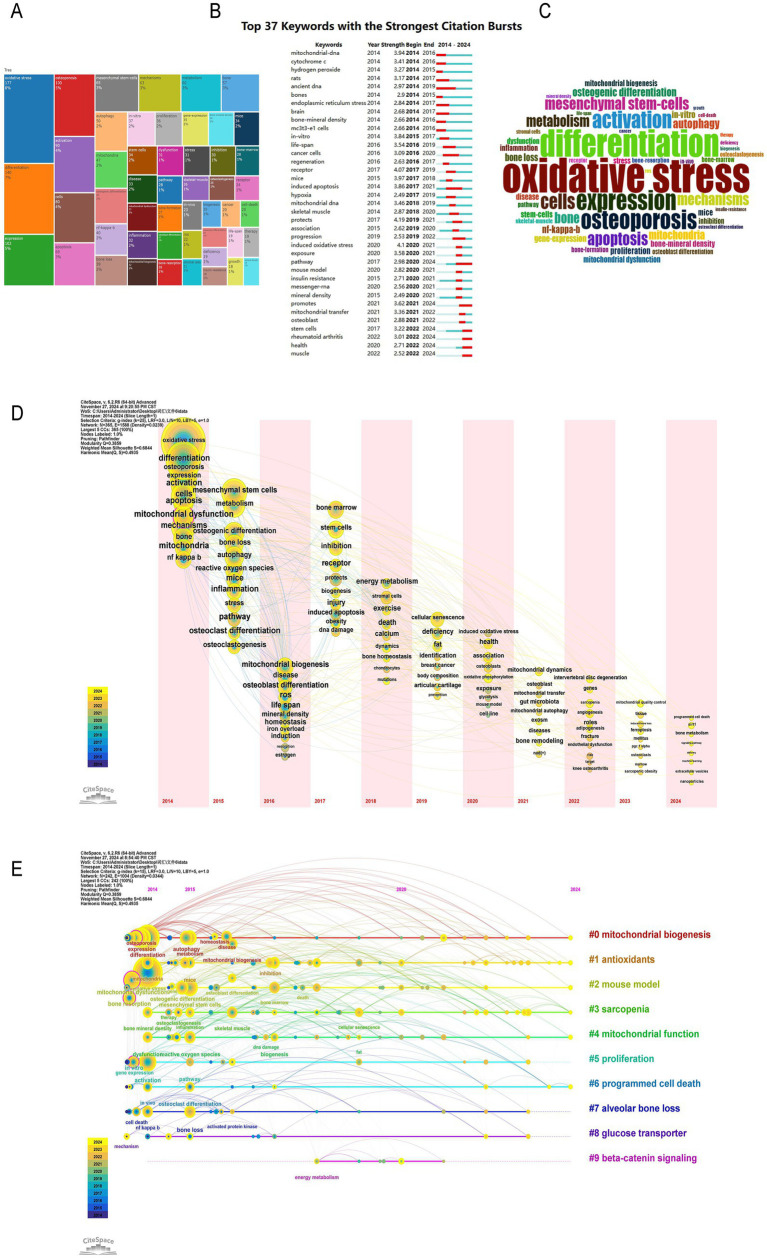
Research hotspots and keywords co-occurrence network. **(A)** Distribution of research topics in a specific field. **(B)** Top 37 keywords with the strongest citation bursts. **(C)** Word cloud of key terms in a specific research field. **(D)** Time zone map of keywords. **(E)** Timeline map of keywords.

Through the statistical analysis of keywords and key terms, we can intuitively show the research hotspots in this field and reflect the research trend. Through the analysis of the frequency of keywords, we found that the keywords with high frequency in this field are oxidative stress (177 times, 8%), differentiation (140 times, 7%), expression (102 times, 5%), osteoporosis (100 times, 5%), activation (90 times, 4%). It can be seen that the role of oxidative stress and cell differentiation in the pathogenesis of osteoporosis has always been a hot spot in this field (Figures 8A,B). Then we use the burst words tool of CiteSpace to analyze the most frequently cited keywords. We found the time distribution of burst words was very close, indicating the field has been in an explosive development stage with rapid research hotspot iteration (Figure 8C). The most popular keywords are mitochondrial transfer (3.36), osteoblast (2.88), stem cells (3.22), promot (3.62), health (2.71) (Figure 8B). The longest duration of the burst word is “induced oxidative stress,” which shows that the relationship between mitochondrial-mediated oxidative stress and osteoporosis has been the focus of scholars for a long time. From the timeline map of keywords, it is clear that research themes such as “mitochondrial biogenesis,” “antioxidants,” “mitochondrial function,” and “programmed cell death” have received widespread attention (Figure 8D). Emerging hotspots in the field of mitochondrial dysfunction and osteoporosis include topics like “ferroptosis” and “SIRT1 protein.” These phenomena reflect the future popular research directions in this field (Figure 8E).

## 4 Discussion

### 4.1 General information

This study retrieved literature on mitochondrial dysfunction and OP from the WoSCC database between 2014 and 2024 for the first time, and visualized it using bibliometric analysis tools CiteSpace and VOSviewer. By statistically analyzing and visualizing highly productive countries, research institutions, authors, journals, references, and keywords from the included 780 papers, we combed through the current status and trends in mitochondrial dysfunction research in osteoporosis over the past decade. The findings show that the annual number of publications in this field has grown overall in the past decade, with a more significant increase since 2016, indicating extensive scholarly attention. The journals with the most references, such as the “Journal of Biological Chemistry, ““Journal of Bone and Mineral Research,” and “Nature,” highlight their authority in this field. Moreover, bibliometric analysis based on Bradford’s Law identified 21 core journals, which are considered the primary choice for researchers in this field. Further statistics show that the annual number of publications in core journals is on the rise, consistent with the overall growth trend in the field, indicating that mitochondrial research in OP has become a hot topic. At the national and institutional levels, we analyzed the number of publications from 60 countries and found that China ranked first with 421 publications, followed by the United States with 173. Analysis of the international cooperation network shows that research distribution in this field is extremely uneven, with a few countries contributing most of the publications. China and the United States have become cooperation centers in this field, while India and Portugal, as emerging countries, are expected to become new research centers in the future. In terms of research institutions, Shanghai Jiao Tong University ranked first with 46 publications, followed by China Medical University and Zhejiang University with 23 each, highlighting China’s core position in this field. Analysis of the cooperation network among research institutions shows that cooperation is close and a mature network has been formed, especially in China. Further correlation analysis indicates that research results in this field are mainly associated with institutions and authors from China and the United States. The complex relationships among countries, institutions, and authors reflect the maturity of international cooperation and academic exchange in this field. This international collaboration has injected strong vitality into the development of the field, promoting its rapid progress.

### 4.2 Research hotspots and frontiers

Through the statistical analysis of keywords and key terms in 780 relevant publications, this study has unveiled the core hotspots and developmental trends in the field of OP research related to mitochondrial dysfunction. Oxidative stress emerged as the most representative research focus, with a keyword frequency of 177 occurrences, accounting for 8% of the total. The formation of multiple dense clusters in the co-citation network further confirmed its central role in the pathogenesis of OP. Additionally, “induced oxidative stress” as the longest-lasting burst term, further highlighted the key role of mitochondrial dysfunction in regulating oxidative stress in the pathological process of OP. Apoptosis, another important mechanism in OP research, had a keyword frequency of 90 occurrences, accounting for 4%. Its co-citation network was significantly associated with literature on oxidative stress and mitochondrial autophagy, suggesting that apoptosis may act as a downstream event of oxidative stress in the regulation of osteocyte fate. In recent years, the research on mitochondrial autophagy has shown a significant upward trend, with a 35% increase in keyword frequency compared to earlier periods. The research primarily focuses on mitochondrial quality control in osteoblasts and osteoclasts, and the interactions of its antioxidant pathways have provided new insights for the treatment of OP. As an emerging mechanism of intercellular communication, mitochondrial transfer has seen a 40% increase in keyword frequency since 2020. On one hand, the transfer of mitochondria from bone lineage cells to myeloid cells can inhibit osteoclast differentiation and regulate hormone-related osteoporosis. On the other hand, abnormal mitochondrial transfer between macrophages and mesenchymal stem cells can affect osteoblast differentiation and exacerbate bone metabolic imbalance. The discovery of these dual mechanisms has highlighted the unique potential of mitochondrial transfer in the regulation of the bone microenvironment.

#### 4.2.1 Impact of oxidative stress on OP

The human redox system is crucial for sustaining life-activities, involving complex electron-transfer processes. At its core is the electron transport chain (ETC) in mitochondria. Specifically, the ETC is located in the mitochondrial inner membrane, responsible for transferring electrons to oxygen to form water, driving ATP synthesis, regulating the cell cycle and cell death, and resisting oxidative damage. However, some electrons may leak and react with oxygen to generate reactive oxygen species (ROS), a key factor in aging and diseases (15). To combat ROS accumulation, cells rely on an antioxidant system composed of enzymatic antioxidants like superoxide dismutase (SOD), catalase (CAT), and glutathione peroxidase (GPX), as well as non-enzymatic ROS scavengers such as glutathione (GSH), ascorbic acid (vitamin C), α-tocopherol (vitamin E), and carotenoids (16, 17). This system not only prevents excessive ROS accumulation but also maintains redox homeostasis and promotes cell protection under oxidative stress. When this balance is disrupted, ROS accumulation triggers oxidative stress, affecting cell fate and tissue homeostasis. In bone metabolism, ROS effects are particularly complex and critical. Physiologically, RANKL binds to its receptor RANK, activating ROS generation in osteoclast precursor cells. As a key intracellular second messenger, ROS regulates OC differentiation, activation, and survival through signaling molecules like Akt, NF-κB, and MAPK, maintaining bone stability and integrity (18). ROS from NADPH oxidase and mitochondrial oxidase are controlled by the antioxidant system (19). However, with age or under certain pathophysiological conditions, mitochondrial dysfunction, increased ROS-related enzyme expression, and antioxidant system imbalance trigger oxidative stress (20, 21). In the bone marrow microenvironment, oxidative stress damages stem cell membranes, proteins, and DNA through excessive ROS, impairing their self-renewal and differentiation potential, inhibiting OB function, and promoting OC activity (22). This disrupts bone formation and resorption balance, inducing OB and bone cell apoptosis, reducing bone density, and causing OP.

In cellular antioxidant defense, multiple signaling pathways maintain redox balance, with the Keap1-Nrf2 pathway being a crucial regulator of detoxification and antioxidant defense (23). Under oxidative or electrophilic stress, Keap1’s cysteine-based redox sensor oxidizes, reducing newly synthesized Nrf2’s nuclear translocation ability, weakening antioxidant defense, and affecting cytokines involved in OB generation (24, 25). In OP patients and ovariectomized mice, significantly enhanced Nrf2 promoter hypermethylation is observed (26), indicating suppressed Nrf2 expression in OP, exacerbating mitochondrial dysfunction in a vicious cycle. Additionally, in H_2_O_2_-induced oxidative stress models, activating NF-κB and reducing Nrf2 expression in mouse mononuclear macrophages significantly increase OC differentiation factor expression, stimulating OC formation (27), proving oxidative stress can regulate bone resorption via the NF-κB pathway. Notably, oxidative stress not only regulates bone metabolism by affecting ROS generation and antioxidant defense balance but also influences bone cell fate through apoptosis, autophagy, etc., with their interaction in bone metabolism gaining attention (28).

#### 4.2.2 Impact of apoptosis on OP

Apoptosis, an orderly self-regulatory death via death receptors and mitochondrial pathways, clears damaged cells, maintains cell homeostasis, promotes tissue renewal and development, and regulates immune and energy metabolism (29, 30). Bone cell apoptosis is key in physiological bone turnover and metabolic bone diseases, balancing OB proliferation and differentiation to determine osteoblast population size, significant for bone remodeling mechanisms and treatment research (31, 32). Increased phosphorylation of p53 and p66shc activates oxidative stress pathways, leading to ROS-induced bone cell apoptosis, suggesting oxidative stress may trigger bone cell senescence and apoptosis (33). Interestingly, apoptotic bone cells are mostly in the oldest cortical areas, not the youngest, possibly because the oldest bone cells are more sensitive to ROS increases (33, 34). Kobayashi et al. (35) found that compared to 2-month-old mice, 24-month-old mice had significantly increased ROS levels. When the mitochondrial SOD2 gene in osteocytes was targeted for deletion, elevated ROS levels were accompanied by a low-bone-mass phenotype, similar to accelerated aging. Fowler et al. (36) observed similar phenomena in cortical bones of aged mice, with bone loss, increased bone porosity, and links to DNA damage, cell senescence, increased senescence-associated secretory phenotype, and heightened RANKL expression in osteocytes. Also, oxidative stress induces apoptosis by increasing ROS and activating apoptotic signaling pathways. FOXO1 maintains bone cell function and skeletal homeostasis by promoting protein synthesis, inhibiting cell cycle arrest, and coordinating stress responses (37). However, in aged mice, elevated ROS levels promote CTNNB1 (β-catenin) transfer from TCF4 to FOXO1-mediated transcription, inhibiting the Wnt/β-catenin signaling pathway, weakening skeletal homeostasis, reducing osteogenesis, and exacerbating bone resorption and fragility (38, 39). Meanwhile, ROS accumulation inhibits METTL3 expression and activates the Wnt/β-catenin signaling pathway, leading to OB apoptosis (30). Another experiment showed that SIRT1 overexpression might enhance FOXO3a transcriptional activity by reducing its acetylation, increasing SOD2 expression, improving osteocyte antioxidant capacity, alleviating oxidative stress-induced bone damage, and countering osteoporosis (40). Additionally, RANKL inhibits FoxO transcription factor activity via Akt-mediated mechanisms, downregulates catalase, and reduces estrogen-induced bone loss (41). These findings suggest that mediating FoxO signaling to inhibit oxidative stress-induced apoptosis could be a potential OP treatment target.

#### 4.2.3 Impact of mitophagy on OP

Autophagy is an intracellular degradation mechanism, which can be divided into selective and non-selective autophagy. Non-selective autophagy mainly randomly degrades intracellular substances to recover nutrients and maintain the basic metabolism of cells. Selective autophagy specifically targets specific organelles or protein aggregates for degradation (42–44). This process plays an important role in maintaining cell homeostasis, responding to nutritional stress, and clearing damaged organelles and proteins. However, excessive ROS can over-activate mitophagy, causing diseases like neurological, cardiovascular, pulmonary, hepatic, and skeletal muscle disorders (45). Mitophagy, a selective autophagy proposed by Lemasters (46) in 2005, removes damaged mitochondria, reducing ROS generation. It is crucial for regulating OB and OC proliferation, differentiation, function, and protecting OB from apoptosis (47). Xiang et al. (48) found that mitochondrial homeostasis disruption and mitophagy inhibition lead to BMSC senescence, blocked osteogenic differentiation, and OP development. OB can protect their survival and differentiation by accelerating autophagy to clear increased mitochondrial fragmentation and swollen mitochondria (49). PINK1 and Parkin are key mitophagy regulators in mitochondrial quality control. In healthy cells, PINK1 is imported into mitochondria and degraded by MPP and PARL, keeping Parkin inactive to inhibit mitophagy (50). When mitochondria are damaged, PINK1 accumulates on the outer membrane and activates Parkin, which ubiquitinates proteins to promote mitophagy and degrade damaged mitochondria (51). Lee et al. (52) found that PINK1 gene knockout inhibits OB differentiation, causing mitochondrial homeostasis disruption, ROS increase, and impaired calcium handling, significantly reducing bone volume and collagen deposition in mouse femurs. In OP patients, PINK1/Parkin-mediated mitophagy reduces plasma AOPPs, inhibits OB apoptosis, and improves bone loss, microstructure damage, and BMD loss (53). Nrf2 upregulates PINK1 expression by binding to the ARE in its promoter, activating the PINK1/Parkin pathway (54, 55), playing a key role in redox balance maintenance and mitophagy promotion. Besides the PINK1/Parkin pathway, Beclin-1/BECN1, p62/SQSTM1, and mTOR pathways also regulate OC mitophagy. Under hypoxia, HIF-1α is crucial for autophagy activation, enhancing WNT/β-catenin signaling for osteoblast senescence and regulating OB differentiation and RANKL expression via HIF-2α, thus inhibiting OB differentiation (56, 57). In summary, mitophagy and its regulatory mechanisms have far-reaching impacts on bone metabolism and cell homeostasis, with complex functions varying by cell type and physiological/pathological conditions.

#### 4.2.4 Impact of mitochondrial transfer on OP

Mitochondrial transfer, via TNT and MV, is a key intercellular communication mechanism (58). As dynamic organelles, transferred mitochondria regulate cascading molecular signals in recipient cells, controlling gene transcription and translation, enhancing cell function, promoting tissue repair, and modulating immune responses. Skeletal mitochondrial transfer mainly involves transfer from mature OBs to OCs and from macrophages to mesenchymal stem cells (59). Mature OBs secrete mitochondria and MDVs to promote osteoprogenitor differentiation, regulated by the CD38/cADPR pathway, involving mitochondrial fragmentation and donut formation (60). Other communication mechanisms remain unclear. Macrophage-to-BMSC mitochondrial transfer directly regulates BMSC osteogenic differentiation and bone homeostasis maintenance (61). Mitochondrial transfer and oxidative stress are closely linked. On one hand, oxidative stress causes mitochondrial dysfunction, triggering RET, increasing ROS production, and further damaging mitochondria and transfer. Cai et al. (61) found that in OP, increased M1 macrophage-to-MSC mitochondrial transfer causes ROS bursts, leading to MSC metabolic remodeling and abnormal osteogenic differentiation, affecting bone homeostasis. On the other hand, oxidative stress increases MERCs, promoting damaged mitochondrial clearance and oxidative stress regulation. Guo et al. (62) confirmed that mitochondrial transfer enhances BMSC proliferation, migration, and osteogenic capacity through aerobic metabolism upregulation, promoting bone defect repair *in vivo*, offering a promising new technology for stem cell therapy optimization. Ding et al. (63) pointed out that impaired mitochondrial transfer from osteogenic cells to mononuclear/macrophages via MIRO1 protein makes bone marrow cells more prone to OC differentiation, promoting bone resorption and loss, while affecting glutathione metabolism and protecting OC precursors from ferroptosis, thus regulating glucocorticoid-induced OP. Glutathione depletion may alleviate GIOP progression. Mitochondrial transfer can deliver antioxidants or enzymes to other cellular regions, clearing excess ROS and mitigating oxidative stress damage. Based on mitochondrial transfer mechanisms, researchers are exploring its potential applications in OP treatment. A novel 3D biomimetic hydrogel scaffold enhances BMSC osteogenic differentiation through immune regulation and macrophage mitochondrial transfer promotion. *In vitro* experiments showed that fluorescent tracing of macrophage mitochondrial transfer promotes BMSC metabolic remodeling, and ALP staining verified osteogenic differentiation ability, demonstrating excellent biocompatibility and accelerated healing capacity (64).

### 4.3 Emerging hotspots

In this study, emerging research hotspots in the field of mitochondrial dysfunction and osteoporosis were identified through multidimensional bibliometric indicators, including keyword frequency, co-occurrence patterns, citation trends, and institutional output. Ferroptosis and SIRT family proteins were recognized as emerging hotspots. Ferroptosis, a form of iron-dependent lipid peroxidation-induced cell death, has seen a significant increase in keyword frequency from a low level in 2018 to 32 occurrences in 2023, with a notable burst strength. Co-citation network analysis revealed that research in this area has formed an independent cluster, involving the intersection of mechanisms related to mitochondrial function regulation, oxidative stress, and bone metabolic imbalance. The research interest in SIRT family proteins was reflected by the increasing keyword frequency and highly cited literature. Family members such as SIRT1 and SIRT3, through their deacetylase activity, regulate mitochondrial antioxidant pathways and apoptosis. The citation frequency of related literature has significantly increased over the past 5 years. The institutional collaboration network showed that research teams from China and the United States have taken the lead in the field of SIRT family and bone metabolism regulation. Their research content includes the protective effects of SIRT1 activators on osteoblast function and the mechanistic dissection of SIRT3 in mitochondrial quality control.

#### 4.3.1 Impact of ferroptosis on OP

Iron is essential for cellular metabolism and function, aiding redox reactions and free radical scavenging. However, iron overload generates ROS via Fenton and Haber–Weiss reactions, inducing ferroptosis, a lipid peroxidation-driven cell death from GPX4 and system xc^−^ downregulation (65, 66). Mitochondria play a key role here. Erastin opens VDAC2/3 on mitochondrial outer membranes, causing iron influx, ROS production, and redox imbalance, leading to ferroptosis (67, 68). This dysfunction exacerbates lipid peroxidation through mechanisms like respiratory chain abnormalities, cysteine depletion, DHODH inhibition, and CoQ10 suppression. Iron metabolism disorders’ impact on OP is a growing research focus (69, 70). Tian’s et al. (71) found that ROS from iron overload induces a RIPK1/RIPK3/MLKL feedback loop, causing OB necroptosis and inhibiting bone formation. Zhang et al. (72) observed in human bones that iron overload makes IRP1 dissociate from IRE-like sequences in the NOX4 locus, activating NOX4 transcription, increasing lipid peroxidation, causing OB mitochondrial dysfunction, ferroptosis, and bone loss. Ferroptosis also promotes OC differentiation and bone resorption, damaging the skeletal microstructure. A study found high iron levels induce osteocyte apoptosis, boost RANKL production, and elevate the RANKL/OPG ratio in osteocytes, enhancing OC differentiation and osteogenesis (73). Hepatocytes-produced hepcidin regulates iron homeostasis by binding to FPN receptors. Zhang et al. (74) found that hepcidin-induced insufficient FPN activation causes iron overload, which deposits in bones, producing ROS, and activating PGC-1β in OCs, leading to OP. Since iron metabolism markers affect type 2 diabetes development, diabetic patients often have iron metabolism disturbances (75, 76). Zhao et al. (77) detected FtMt expression and ferroptosis in bone tissues of T2D rats. In high sugar and high-fat diabetic rats, weakened SLC7A11 and GPX4 expression activate the METTL3/ASK1-p38 pathway, inducing OB ferroptosis, inhibiting OB differentiation and mineralization (78). Yang et al. noted that ferroptosis causes bone cell death in DOP through lipid peroxidation and HO-1 activation, with targeting ferroptosis or HO-1 offering effective DOP improvement strategies. Li et al. (79) developed curcumin and tFNA-based nanoparticles to inhibit ferroptosis by activating the NRF2/GPX4 pathway, promoting BMSC osteogenic differentiation in diabetic microenvironments, reducing trabecular loss, and increasing bone formation, showing potential for DOP treatment. Estrogen withdrawal disrupts iron metabolism, making it harder for postmenopausal women to excrete iron. Postmenopausal estrogen decline is linked to increased iron accumulation, which is associated with a higher incidence of clinical postmenopausal OP (69, 80). Studies show that osteocyte ferroptosis, regulated by the Nrf2 pathway, affects RANKL expression via Dnmt3a-mediated DNA methylation of the RANKL promoter, influencing OC formation and contributing to postmenopausal osteoporosis (PMOP) (81). Research indicates that chelating excess iron, activating the KEAP1/NRF2/HMOX1 pathway, and inhibiting ferroptosis via the SLC7A11/GSH/GPX4 pathway can suppress MSCs ferroptosis, promote osteogenesis, and offer new PMOP treatment strategies (82).

#### 4.3.2 Impact of SIRT family on OP

The SIRT family (sirtuins), NAD^+^-dependent deacetylases, are involved in multiple biological processes, including cell metabolism, oxidative stress, gene expression regulation, the cell cycle, apoptosis, and senescence, making them key targets against age-related diseases (83, 84). Recently, their role in bone metabolism has garnered attention. SIRT1 reduces bone resorption by inhibiting NF-κB signaling in osteoclasts and promotes osteoblast differentiation and function via RUNX2 deacetylation, thus maintaining bone homeostasis (84–86). It also regulates oxidative stress, protects osteoblasts from oxidative stress-induced apoptosis by interacting with FOXO3A, and enhances their antioxidant defense (87). Resveratrol activates SIRT1 to boost autophagy in osteoporotic rat osteoblasts, improving their survival and function. However, SIRT1 levels and activity in bone progenitor cells decline with age. This decline inhibits Wnt signaling by preventing FoxO transcription factors from capturing β-catenin, reducing bone cell proliferation and increasing apoptosis, thereby exacerbating bone loss (88). Activating SIRT1 can improve osteoblast function under pathological conditions. Clinical studies show that nicotinamide riboside and glucosamine supplements activate SIRT1, helping maintain bone mass in conditions like estrogen withdrawal and glucocorticoid therapy (89). Yang et al. (90) indicates that the SIRT1 agonist resveratrol regulates the SIRT1/PGC1α axis to regulate bone metabolism and combat osteoporosis caused by vitamin D deficiency. Under hypoxia, SIRT1 binds to protein kinase B via lysine deacetylation, inhibits caspase 3 and 9 activity, and protects MC3T3-E1 osteoblasts from apoptosis, enhancing cell viability (91). Clinically, reduced SIRT1 in adult femurs affects osteoporosis-related protein expression in the femoral neck and can lead to osteoporotic hip fractures (92). Moreover, SIRT1 influences ferroptosis by affecting redox balance, iron metabolism, and lipid metabolism. Activating the SIRT1/Nrf2/GPx4 pathway can suppress ferroptosis and protect cells from oxidative stress (93). Other SIRT family members also play roles in osteoporosis. For example, increased hepatic SIRT2 expression promotes osteoporosis in aged mice and humans, while SIRT2 deficiency reduces bone resorption by upregulating LRG1 in sEVs, with LRG1 levels positively correlated with bone density (94). SIRT2 also promotes BMM-to-OC differentiation by regulating c-Fos and NFATc1, influencing age-related bone loss (95). Sirt3 deficiency impairs oxidative phosphorylation, increases ROS in OCs, reduces autophagy, and promotes OC maturation via a PINK1-independent mechanism, leading to bone loss in women (96).

### 4.4 Clinical application potential

Based on long-term systematic research on the pathogenesis of OP related to mitochondrial dysfunction, the core hotspots and emerging research areas in this field have gradually shifted from mechanism exploration to therapeutic strategy development. Zhang et al. (97) demonstrated that C1q tumor necrosis factor-related protein 3 (CTRP3) can protect OB from oxidative stress injury and promote bone formation by activating the AMPK/SIRT1/Nrf2 signaling pathway, thereby inhibiting the progression of OP. The team led by Wang et al. (98) also pointed out that SIRT1 can enhance the transcriptional activity of Nrf2 through deacetylation, thereby activating the expression of downstream antioxidant genes. This interaction is crucial for protecting cells from oxidative damage. Ling et al. (99) successfully reversed mitochondrial dysfunction caused by Sirt3 deficiency and excessive bone resorption mediated by estrogen deficiency, as well as bone loss in mice. These findings indicate that therapeutic strategies targeting oxidative stress hold significant research importance and potential application value. Studies have shown that vitamin K2 (VK2) can restore bone mass and enhance the expression of SIRT1, GPX4, and osteogenic markers in the distal femur by inhibiting bone loss and ferroptosis mediated by long-term high glucose (HG). VK2 achieves this by activating the AMPK/SIRT1 signaling pathway to ameliorate type 2 diabetes-associated osteoporosis (T2DOP) and suppress ferroptosis (100). Additionally, a novel active vitamin D derivative, eldecalcitol (ED-71), has been shown to prevent bone loss caused by osteoporosis by inhibiting cellular senescence of BMSCs in ovariectomized rats through the regulation of the SIRT1-Nrf2 signaling pathway, demonstrating promising efficacy in *in vivo* experiments (101). However, most current studies are based on cell experiments and animal models, lacking large-scale clinical trials in humans to verify the effectiveness and safety of these interventions for treating osteoporosis and osteonecrosis in different populations (102). Challenges remain in translating these research findings into clinical applications. However, clinical studies have confirmed that zoledronic acid and melatonin can inhibit oxidative stress via the Sirt3/SOD2 pathway, accelerate osteogenesis of BMSCs, and delay the progression of OP, demonstrating potential for treating bone loss (103, 104). These preliminary clinical results not only provide strong support for therapeutic strategies targeting oxidative stress but also point the way for the development and clinical application of related drugs in the future. Some plant-derived peptides, which can be obtained from natural and climate-friendly sources, are increasingly attracting attention in new drug development due to their various biological activities, including antioxidant properties (105). For example, *Ginkgo biloba* leaf extract can improve OP by influencing the SIRT3/NF-κB axis in OC and promoting M2 polarization in macrophages (106). Salvianolic acid A (SAL-A) protects osteoblasts from H₂O₂-induced oxidative stress by scavenging free radicals, enhancing osteoblast viability, mineralization, and differentiation, and inducing oxidative stress in rat osteoblasts (107). Curculigoside, a natural product widely found in plants, has shown significant positive effects in animal models by inhibiting inflammatory responses, antagonizing oxidative stress, and regulating various signaling pathways, as well as modulating the differentiation and function of osteoblasts (108). *Lycium barbarum* polysaccharides (LBP), a potential bone-enhancing agent, can improve cell viability and osteogenic differentiation. It alleviates the inhibitory effect of cadmium (Cd) on osteogenesis in BMSCs by stimulating the autophagy process and promoting the formation of autophagosomes and autolysosomes (109). In recent years, several novel therapeutic approaches have shown significant therapeutic potential in the field of OP and related bone defect repair. Kang et al. (110) developed a calcium phosphate cement (CPC) modified with α-ketoglutarate (α-KG) polyester microspheres (CPC/α-KG). This material enhances osteogenic differentiation and biomineralization capabilities by inhibiting inflammation and oxidative stress via the PI3K/AKT pathway, thereby improving the osteogenic microenvironment. Chen et al. (111) constructed a microenvironment-responsive coordination nanoparticle composed of salvianolic acid B, catechol-conjugated chitosan, and Ca^2+^, which was immobilized on the surface of titanium implants. The nanoparticles remain stable under physiological conditions and can moderately regulate immune responses. However, under acidic and oxidative pathological conditions, they rapidly decompose to achieve ROS scavenging, anti-inflammatory effects, and bone induction, thereby remodeling the pathological microenvironment into a regenerative microenvironment. Additionally, nanovesicles extracted from carrot juice by the extrusion method remain structurally stable at pH 7.4 but aggregate at pH 4.0. These vesicles can passively target osteoporotic bone and effectively reduce bone loss by alleviating oxidative stress, restoring mitochondrial function, and promoting osteoblastogenesis (112). The emergence of these novel therapeutic approaches not only enriches the treatment options for osteoporosis and related bone defect repair but also provides new ideas and directions for the development of more efficient and targeted therapeutic methods in the future.

### 4.5 Limitations

Using bibliometrics, this study performed an in-depth analysis of the relationship between mitochondrial dysfunction and osteoporosis during the period from 2014 to 2024. It emphasizes the critical role of mitochondrial dysfunction in the pathogenesis of osteoporosis and offers a novel perspective for future prevention and treatment strategies. Nevertheless, certain limitations exist in this study. The analysis was restricted to English-language literature within the WoSCC core collection, potentially overlooking relevant studies in other languages or databases. Additionally, the research predominantly relied on bibliometric methods, which are effective for macro-level analysis of academic output and research trends but may lack qualitative assessments to complement the quantitative findings. While the study integrates knowledge from multiple disciplines, such as biology and medicine, it may fall short in achieving deeper interdisciplinary collaboration. Consequently, its direct implications for clinical application are somewhat limited. Future research should aim to incorporate literature from diverse languages and databases to achieve a more comprehensive research framework. Furthermore, integrating qualitative analysis methods could enhance understanding of key issues and provide clearer guidance for future research directions. Based on the established connection between mitochondrial dysfunction and osteoporosis, we propose the development of innovative therapeutic strategies.

## 5 Conclusion

This study employed bibliometric analysis to systematically investigate the research trends in mitochondrial dysfunction and OP over the past decade for the first time. Overall, the number of publications in this field has shown a steady increase and has fostered extensive global collaboration networks. Oxidative stress, apoptosis, mitophagy, and mitochondrial transfer are identified as current research hotspots, while ferroptosis and the SIRT family of proteins have emerged as promising new directions for future research. This study systematically summarized and analyzed the research hotspots and emerging trends in this field, which can help clinicians and researchers better grasp the future research directions and provide theoretical and practical guidance for the prevention and treatment of OP.

## Data Availability

The original contributions presented in the study are included in the article/supplementary material, further inquiries can be directed to the corresponding author.
